# Controlled oxidation of graphite to graphene oxide with novel oxidants in a bulk scale

**DOI:** 10.1007/s11051-012-1248-z

**Published:** 2012-10-30

**Authors:** Malgorzata Wojtoniszak, Ewa Mijowska

**Affiliations:** Institute of Chemical and Environment Engineering, West Pomeranian University of Technology, Pulaskiego 10, 70-322 Szczecin, Poland

**Keywords:** Graphite, Chemical exfoliation, Graphene oxide

## Abstract

In this study, a novel method of graphite chemical exfoliation to create graphene oxide (GO) is reported. Here, new oxidants were examined: a mixture of perchloric and nitric acids and potassium chromate. Furthermore, an effect of oxidation time, temperature of oxidation, and ultrasonication on graphite exfoliation degree was investigated. The obtained GOs were next reduced with glucose, used as a reducing agent. Detailed analysis of the materials indicated that when graphite was oxidized for 24 h at 50 °C, 5-layered graphene was prepared. An effect of sonication process was also examined, and it was found to enhance the exfoliation to bilayer graphene. Furthermore, when time and temperature were increased to 48 h and 100 °C, respectively, graphite was exfoliated to single-layer graphene. Therefore, it is believed that the proposed route can be applied for the preparation of graphene or few-layered graphene with defined number of layers upon the process parameters optimization and in a bulk scale. The materials were characterized with atomic force microscopy, Fourier-transform infrared spectroscopy, Raman spectroscopy, and X-ray diffraction.

## Introduction

Graphene shows excellent properties, such as high intrinsic carrier mobility (200,000 cm^2^/V^3^ s), superior thermal conductivity, and excellent mechanical strength and elasticity (Bolotin et al. 2008; Balandin et al. 2008; Lee et al. 2008). Since graphene was isolated in 2004 by Geim and coworkers (2004) using a scotch tape method, there have been many processes developed to produce graphene. Although the mechanical exfoliation of graphite leads to production of high-quality and high-mobility graphene flakes, this method is a time consuming process limited to a small scale production. One of the methods is thermal decomposition of SiC in temperature range between 1,000 and 1,500 °C. Here, Si sublimate from the silicon carbide and leave behind a carbon-rich surface (Hass et al. 2008). This method requires controlling the number of graphene layers, repeatability of large area growths, and interface effects with the SiC substrate (Choi et al. 2010). The second method of graphene synthesis is chemical vapor deposition (CVD). This process is promising in large scale production of graphene. Many papers concerning CVD growth of graphene using different catalysts (Ni, Cu, ZnS, Fe) have been reported (An et al. 2011; Wei et al. 2009; Li et al. 2009; Reina et al. 2009; Obraztsov 2009). Graphene can be also synthesized via chemical exfoliation of graphite, where interlayer van der Waals forces are eliminated: chemical derivatization, intercalation, thermal expansion, the use of surfactants, and oxidation–reduction (Chakraborty et al. 2008; Lotya et al. 2009; Lee et al. 2008). The most common route to chemically exfoliate graphite is the use of strong oxidants to produce graphene oxide in a first stage. Graphite oxide was first prepared by B.C. Brodie, where it was treated with a mixture of potassium chlorate and nitric acid (Brodie 1859). Later, Hummers and Offeman (1958) used a mixture of sulfuric acid, sodium nitrate, and potassium permanganate to oxidize graphite. Recently, many papers reporting a modification of the Hummers method have been published. For instance, Marcano et al. (2010) used a mixture of sulfuric and orthophosphoric acids and potassium permanganate in the oxidation process, and found it improved an efficiency of the oxidation. This process is still widely investigated for two reasons: (1) it gives an opportunity to produce large scale graphene or few-layered graphene, (2) it has potential to provide the samples with controlled number of graphene layers upon the process parameters optimization.

In this study, novel oxidants of graphite, toward its chemical exfoliation, were examined: a mixture of perchloric and nitric acids and potassium chromate. Furthermore, an effect of oxidation time, temperature of oxidation and ultrasonication in exfoliation degree were investigated. The obtained materials were characterized with TEM, FT-IR, Raman spectroscopy, and XRD.

## Experimental

### Materials

Graphite was purchased from Alfa Aesar (synthetic, 99.9995 %, 325 mesh). Perchloric acid, nitric acid, hydrochloric acid, and ethanol were obtained from Chempur. K_2_CrO_4_ was bought from POCH.

### Methods

Here, three types of graphene oxides (GOs) were prepared. In the first procedure of GO1 synthesis, 1 g of graphite was dispersed in a mixture of perchloric and nitric acids (350 mL, 4:3—volume ratio), and next, K_2_CrO_4_ (6 g) was added. The mixture was then heated to 50 °C and reaction was operated for 24 h. The obtained mixture was next filtrated through polycarbonate (PC) membrane (Whatman 0.2 μm) and washed three times with ethanol (200 mL) and 10 % hydrochloric acid (200 mL) to remove residual metal ions, and finally with distilled water until pH of the solution was 7. Finally, the material was dried in air at 100 °C for 24 h.

In the second method, prior heating a mixture of graphite, perchloric acid, nitric acid, and K_2_CrO_4_, an ultrasonication process was performed for 6 h at room temperature. After oxidation process, the purification, filtration, and drying steps were realized as in the first procedure, and finally GO2 was obtained.

In the third route, the time and the temperature of oxidation process were increased to 48 h and 100 °C, respectively. The sonication, purification, filtration, and drying steps were carried out as in the second procedure to obtain GO3. After the purification process of the each graphene oxide, the content of impurities was determined with thermogravimetric analysis. It was estimated that GO1, GO2, and GO3 contain 0.11, 0.19, and 0.08 wt% of contaminants, respectively (data are not presented here).

Reduced graphene oxide (RGO) was synthesized with glucose using as a reducing agent (Zhu et al. 2010). In a typical procedure, graphene oxides (GO1, GO2, and GO3) were separately dispersed in 50 mL of water (0.5 mg/mL) and ultrasonicated for 2 h. Next, 80 mg of glucose was added to each homogeneous GO dispersion, and the mixtures were stirred for 30 min. Then, 40 μL of ammonia solution was added and reactions were heated to 95 °C, stirring simultaneously for 2 h. Next, the each reaction mixture was filtered through PC membrane (0.2 μm Whatman). The obtained solid material was then washed with water and ethanol (3 times). Finally, the products (RGO1, RGO2, and RGO3) were dried in air at 100 °C for 24 h.

The morphology of the obtained materials was characterized via atomic force microscopy (Nanoscope V MultiMode 8, Bruker). The measurements were done in air under ambient conditions. Raman measurements were performed on an In-Via Raman microscope (Renishaw) with excitation laser wavelengths of 785 nm. Raman spectra were obtained from individual flakes deposited on SiO_2_/Si wafer (300 nm SiO_2_) (Liana et al. 2010). The crystallographic structure of the samples were characterized by XRD analysis (X’Pert PRO Philips diffractometer) using a CuKα radiation. FT-IR absorption spectra were recorded on Nicolet 6700 FT-IR Spectrometer.

## Results and discussion

**Fig. 1:**
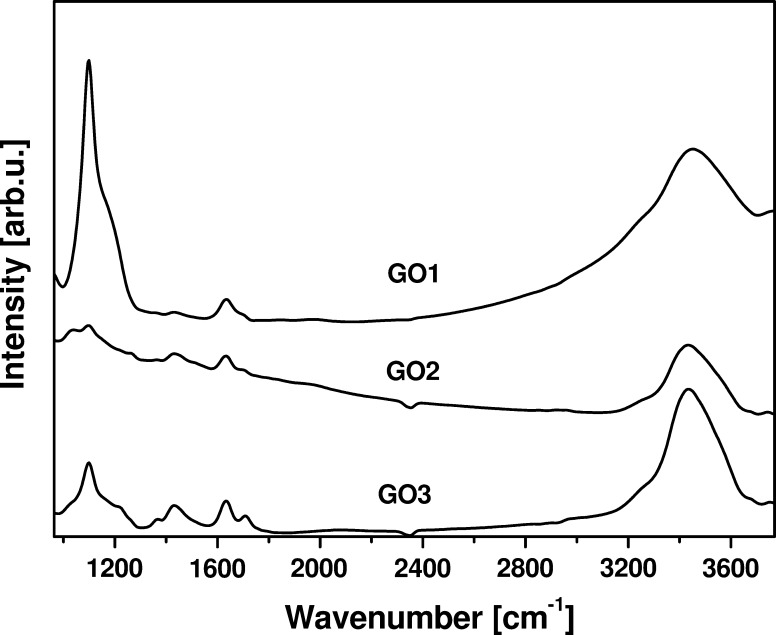
FT-IR spectra of GO1, GO2, and GO3

In this study, novel oxidants were used to create chemical exfoliation of graphite. In order to examine the efficiency of oxygen-containing functional groups introduction to carbon lattice, FTIR spectroscopy was used. Figure 1 presents FTIR spectra of GO1, GO2, and GO3. At each spectrum, several typical modes corresponded to oxygen-functional groups are detected. Here, stretching vibration modes of C–O bonds arise at 1,099 cm^−1^. The peaks between 1,190 and 1,380 cm^−1^ are related with C–OH stretching vibration (Xu et al. 2008). Deformation vibration modes of O–H groups are observed at 1,431 cm^−1^ (Stankovich et al. 2006). The bands appeared at 1,635 cm^−1^ correspond to adsorbed water molecules indicating hydrophilic properties of the material (Paredes et al. 2008). At approximately 1,709 cm^−1^ C=O stretching vibration of carboxyl groups are detected. The peaks at around 3,430 cm^−1^ arise from O–H stretching vibration (Wojtoniszak et al. 2012). FTIR spectroscopy confirms successful oxidation. Furthermore, the intensities of the modes between 1,230 and 1,740 cm^−1^ increased in order of GO1, GO2, and GO3 which suggests an increase of the oxidation efficiency.

**Fig. 2:**
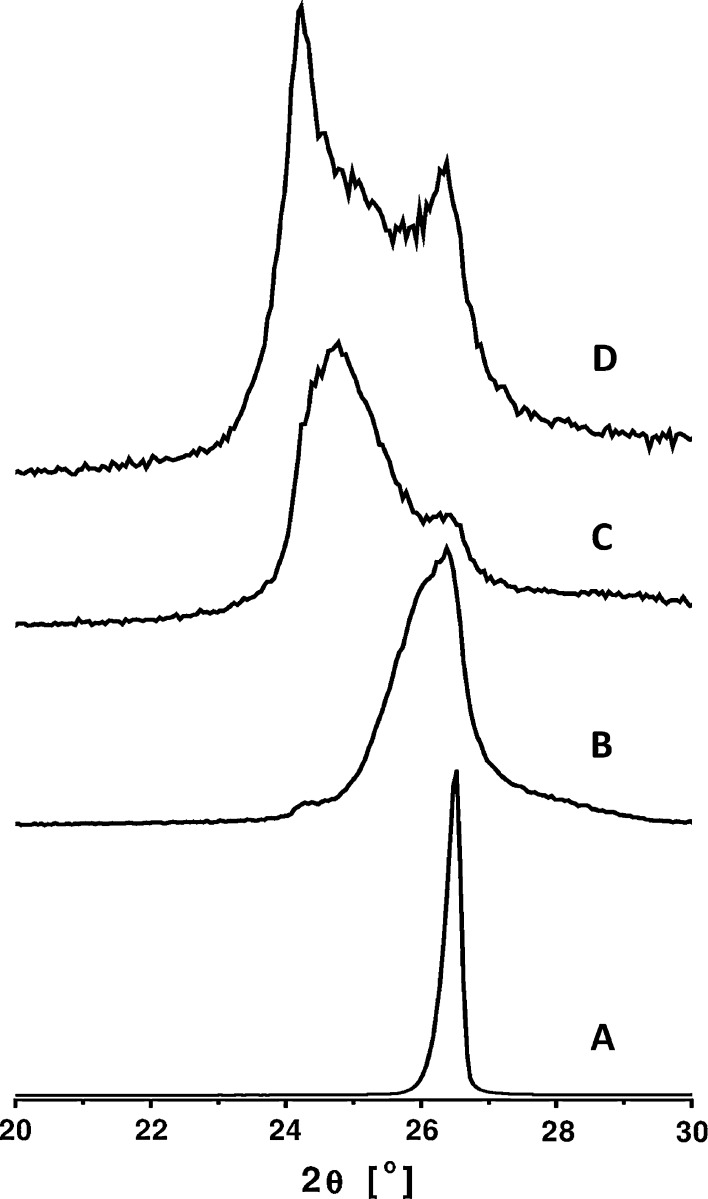
XRD patterns of graphite (**a**), GO1 (**b**), GO2 (**c**), and GO3 (**d**)

For further analysis XRD was used to monitor chemical exfoliation of graphite in a bulk scale. Figure 2 shows XRD patterns of graphite, GO1, GO2, and GO3. XRD pattern of graphite is dominated by single peak at 26.48° corresponding to (002) plane (Liana et al. 2010). After oxidation process, the peak started to vanish, and new peaks arose at 24.28°, 24.78°, and 24.23° in XRD patterns of GO1, GO2, and GO3, respectively. On the basis of Bragg’s law, the interlayer spacing (*d*_001_) of GO1, GO2, and GO3 is 3.7, 3.6, and 3.7 Å, respectively. It is enhanced compared to that of graphite (3.4 Å) (Fan et al. 2011). In GO2 and GO3 XRD patterns, more distinct decay of the peak at 26.48° is observed. It suggests an enhanced exfoliation of graphite when oxidation was performed with ultrasonication treatment and when a time and a temperature was increased, also confirming the enhanced oxidation effect.

**Fig. 3:**
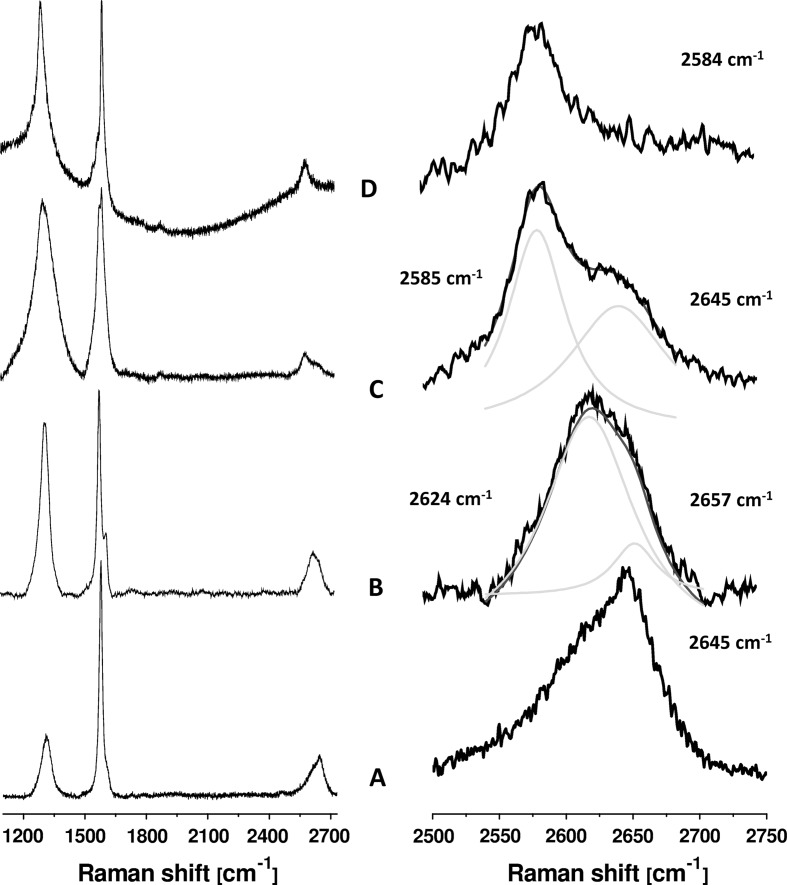
Raman spectra of graphite (**a**), RGO1 (**b**), RGO2 (**c**), and RGO3 (**d**)

**Fig. 4:**
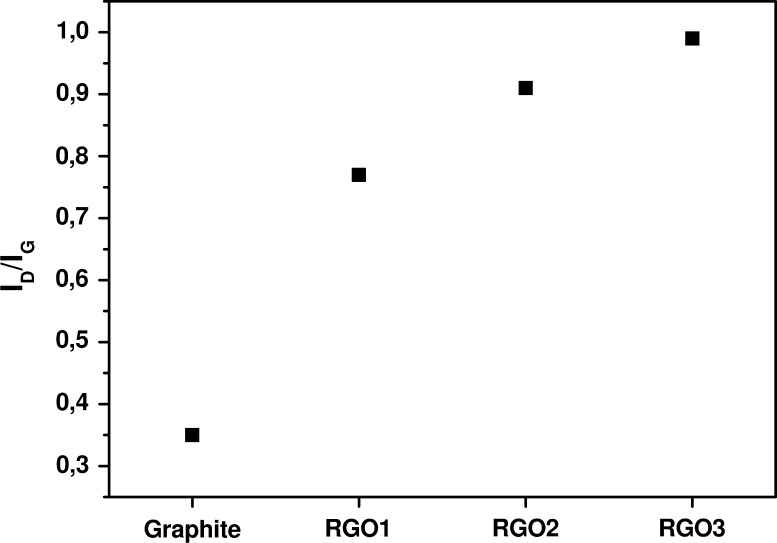
Relative defects content in graphite, RGO1, RGO2, and RGO3 measured as ratio of D and G peaks intensities (*I*_D_/*I*_G_)

In order to determine the number of graphene layers, the samples underwent the reduction process. For reduced graphene oxide (RGO) preparation, glucose was used as a reducing agent. The obtained materials (RGO1, RGO2, and RGO3) were analyzed with Raman spectroscopy to characterize their structure, in terms of number of graphene layers and lattice defects. Figure 3 presents Raman spectra of graphite (A), RGO1 (B), RGO2 (C), and RGO3 (D). All spectra exhibit 3 typical modes related to graphite-like structure: a G band, a D band, and a 2D band (Fig. 3, left panel). The G mode at approximately 1,580 cm^−1^ originates from the in-plane vibration of sp^2^ carbon atoms and is a doubly degenerate phonon mode (*E*_2g_ symmetry) at the Brillouin zone center (Li et al. 2007). The D band at roughly 1,320 cm^−1^ is a breathing mode of *A*_1g_ symmetry involving phonons near the *K* zone boundary (Wilson et al. 2009). The 2D band arises from a two phonon double resonance Raman process (Ferrari and Robertson 2000). The 2D band is a key mode in identification of number of graphene layers. According to its position, shape and intensity, a thickness of graphene may be determined (Ferrari and Robertson 2000; Ni et al. 2008). Charlier et al. (2008) plotted the evolution of the 2D band as a function of layers in single-, bi-, and few-layer graphene. Briefly, a 2D band of bi- and few-layer graphene has 2 components 2D_1_ and 2D_2_. Increasing the number of layers leads to a significant increase of the relative intensity of the 2D_2_ peak and blue shift of the 2D peak. However, a 2D peak of single-layer graphene is composed of a single 2D peak, positioned at roughly 2,600 cm^−1^. Therefore, a Lorentzian fitting of the RGOs spectra was performed and is presented in Fig. 3 (right panel). Here, 2D band of RGO1 differs from 2D band of graphite, and has two components: 2D_1_ and 2D_2_ with maximal intensity at 2,624 and 2,657 cm^−1^, respectively. It may be attributed to 5-layered graphene. The 2D_2_ peak of RGO2 decreases in comparison to RGO1. Furthermore, the 2D peak of RGO2 shifts to lower frequencies. These observations suggest the enhanced exfoliation of graphene when sonication was utilized leading to creation of bilayer graphene. It is clearly seen that 2D_2_ peak of RGO3 vanishes, and single 2D peak appears at 2,584 cm^−1^ attributed to single-layer graphene (Ferrari et al. 2006). It is known that a ratio between the intensities of the D and the G bands (*I*_D_/*I*_G_) determines relative defect content in the carbon lattice (Jorio et al. 2011). Therefore the *I*_D_/*I*_G_ ratios of RGOs spectra were calculated and results are presented in Fig. 4. It is shown that the *I*_D_/*I*_G_ ratio increases in order of RGO1, RGO2, and RGO3. This is related to the formation of vacancies and defects in carbon lattice, such as five- and seven-membered carbon rings, during the reduction process (Schniepp et al. 2006). This means that introduction of more oxygen-functional groups during the oxidation results in formation of more defects and vacancies in the final product—graphene.

**Fig. 5:**
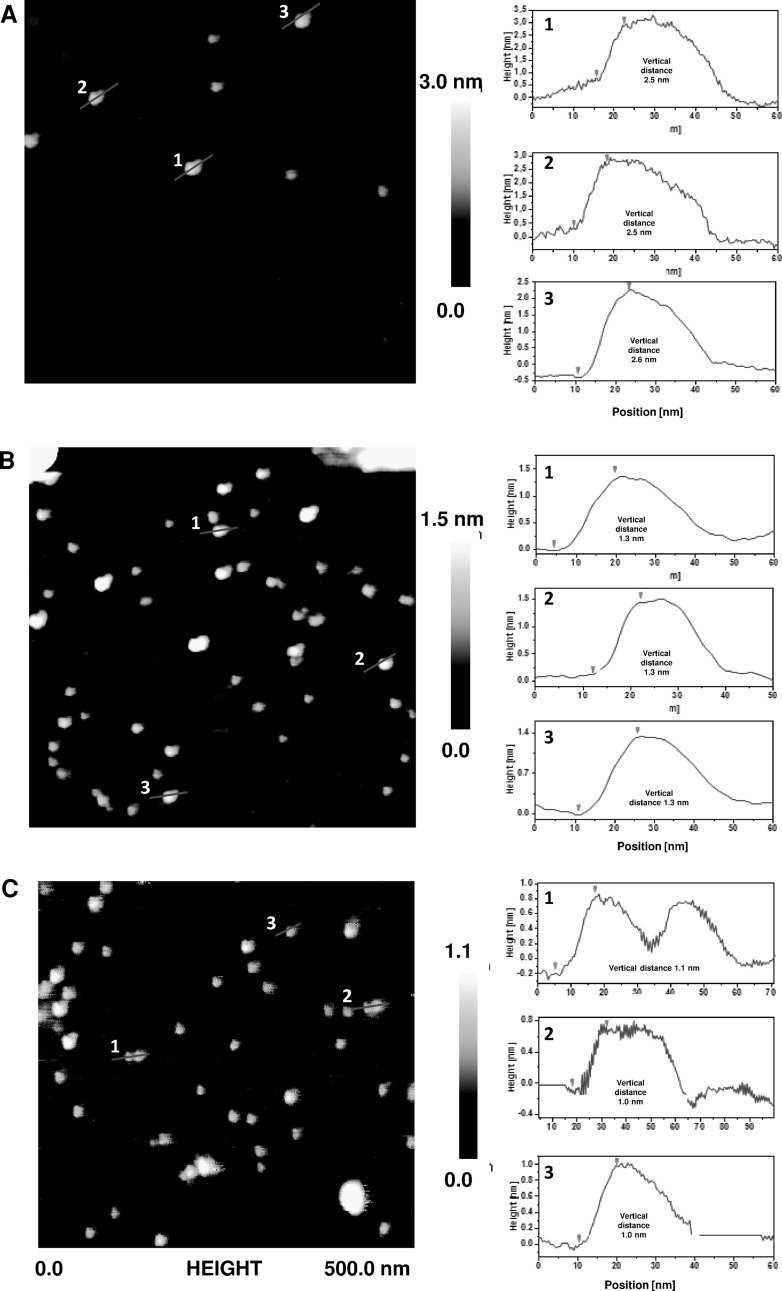
AFM images (*left*) and height profiles (*right*) of a 5-layered graphene of RGO1 (**a**), bilayer graphene of RGO2 (**b**), and single-layer graphene of RGO3 (**c**). Scan size 500 × 500 nm

To confirm the morphology of RGO1, RGO2, and RGO3, atomic force microscopy was applied. Figure 5 presents topography and height profiles of the prepared nanomaterials. The sizes of graphene flakes ranged from 30 to 70 nm in the each sample. The thickness of RGO1, RGO2, and RGO3 was 2.5–2.6, 1.3, and 1.0–1.1 nm, respectively. It was already reported that the thickness of a single-layer graphene on a substrate SiO_2_/Si with approximately 1 nm rms roughness, would be 0.8–1.2 nm. The enhanced thickness of monolayer graphene may be attributed to the interaction between the sample and the tip (Gupta et al. 2006; Nemes-Incze et al. 2008). In bi- or few-layered graphene, the thickness arises with additional layer and therefore, adding the expected 0.35 nm height corresponding to the native van der Waals inter-layer distance (Soldano et al. 2010). It was concluded that RGO1, RGO2, and RGO3 were composed of 5-layered, bilayer, and monolayer graphene, respectively (Li et al. 2008a, b). This observation is in full agreement with Raman response of the samples.

## Conclusions

In summary, novel method of chemical exfoliation of graphite was presented. Here, novel oxidants were examined: a mixture of perchloric and nitric acids and potassium chromate, and an effect of oxidation time, temperature of oxidation, and ultrasonication in exfoliation degree were investigated. The presented methodology leads to creation of graphene with controlled number of layers: single-, bi-, and 5-layered graphene in a bulk scale.
